# The effect of dietary fiber based on fermentability and viscosity on the gut microbiome composition in chronic kidney disease: a systematic review of experimental and clinical trials

**DOI:** 10.21203/rs.3.rs-10462835/v1

**Published:** 2026-07-27

**Authors:** Seyedeh Nooshan Mirmohammadali, Claudia Carrillo, Jason B. Reed, Brandon M. Kistler, Hannah E. Wilson, Bruce Hamaker, Sharon M. Moe, Annabel Biruete

**Affiliations:** Purdue University West Lafayette; Iowa State University; Purdue University West Lafayette; Purdue University West Lafayette; Indiana University; Purdue University West Lafayette; Indiana University; Purdue University West Lafayette

**Keywords:** Chronic kidney disease, gut microbiome composition, alpha and beta diversity, dietary fiber, fermentability and viscosity

## Abstract

**Background:**

Chronic kidney disease (CKD) is associated with alterations in gut microbial composition and diversity. Accordingly, dietary fiber has emerged as a promising microbiome-targeted intervention. However, the influence of fiber fermentability and viscosity remains largely understudied.

**Objective:**

To systematically evaluate the effects of dietary fiber supplementation interventions, classified by viscosity and fermentability, on gut microbial composition in CKD across human and animal studies, and to determine whether these fiber properties influence gut microbiota composition.

**Methods:**

A systematic search of PubMed, Embase, CINAHL, and Cochrane Library (through February 2026) identified randomized controlled trials and controlled animal studies assessing isolated dietary fiber in CKD. Eligible studies reported gut microbiota composition. Risk of bias was assessed with ROB-2 and SYRCLE, and an overall level of evidence (strong, moderate, weak, or inconclusive) was assigned based on study quality and consistency of findings. Alpha and beta diversity, as well as bacterial taxa changes, were then reported according to these evidence rankings.

**Results:**

Twenty-two studies (9 human, 13 animal) were included, comprising 236 participants and 274 animals with CKD. Dietary fiber supplementation demonstrated limited and inconsistent effects on gut microbiota composition in human studies. Inulin and resistant starch were associated with weak evidence of changes in beta diversity, but most taxa across phylum and genus levels remained unchanged, often supported by strong or weak evidence depending on the taxonomic level. Modest increases were observed in a small number of genera, including *Verrucomicrobia, Akkermansia, Clostridium* by inulin, Oscillospiraceae UCG-002, and *Subdoligranulum* by resistant starch type 2 in human studies, which were not consistent across animal studies. Beta-glucan showed no significant effects on alpha or beta diversity or microbial composition. In contrast, weak evidence from animal studies suggested that beta-glucan may increase alpha diversity.

**Conclusion:**

Isolated dietary fiber interventions were associated with variable changes in gut microbiota composition in CKD, with limited consistency across human and animal studies, and no clear pattern based solely on fermentability or viscosity. Future studies should use standardized sequencing approaches, harmonized taxonomic reporting, and detailed characterization of fiber properties to clarify whether specific fibers reproducibly modulate microbial community structure.

This trial was registered at PROSPERO as CRD42023483468.

## Introduction

Chronic kidney disease (CKD) is a progressive condition characterized by sustained reductions in kidney function and affects approximately 10–15% of adults worldwide (1). Progressive nephron loss impairs the kidney’s ability to maintain metabolic, endocrine, and electrolyte homeostasis, resulting in the accumulation of uremic solutes and disturbances in mineral and bone metabolism, acid–base balance, hormonal regulation, and other alterations (2). Beyond these established complications, CKD is increasingly associated with alterations in the intestinal microbial ecosystem or gut microbiome (3, 4). The uremic state, together with dietary modifications, medication use, and prolonged colonic transit time, may alter the gastrointestinal environment and exert selective pressures on resident microbial communities (4, 7).

Beyond the well-established metabolic and physiological consequences of CKD, increasing attention has focused on the gut microbiome as a potential contributor to disease progression and CKD-related complications (4, 7). The gut microbiome comprises the collective community of microorganisms, including bacteria, archaea, fungi, and viruses, that inhabit the gastrointestinal tract and interact closely with host metabolic, immune, and physiological processes (8, 9). Importantly, the biological effects of the gut microbiome extend beyond microbial composition alone and are largely mediated through microbial metabolic activity and the production of bioactive compounds that influence host physiology (10). CKD is closely linked to alterations in the gut microbiome through the bidirectional gut–kidney axis. As kidney function declines, urea accumulates in the blood and diffuses into the intestinal lumen, creating a gut environment that favors proteolytic fermentation over saccharolytic fermentation (11). This shift may promote the growth of urease-producing and protein-fermenting bacteria, including members of Enterobacteriaceae and Streptococcus, while reducing beneficial short-chain fatty acid–producing taxa, such as *Roseburia*, *Blautia*, and *Faecalibacterium* (12). These microbial changes may contribute to intestinal inflammation and disruption of epithelial tight junctions, increasing gut permeability. As a result, microbial metabolites and gut-derived uremic toxins can more readily enter the circulation. In particular, proteolytic fermentation of amino acids leads to the production of protein-bound uremic toxins, including indoxyl sulfate, p-cresyl sulfate, and trimethylamine N-oxide (13). Accumulation of these metabolites may further contribute to systemic inflammation, cardiovascular complications, kidney fibrosis, and CKD progression. Given the strong influence of diet on microbial ecology, dietary fiber has emerged as a promising strategy for modulating gut microbiome composition (14).

Advances in high-throughput sequencing technologies, particularly 16S rRNA gene sequencing and shotgun metagenomic sequencing, have transformed our understanding of the gut microbiome by enabling comprehensive characterization of microbial community structure, taxonomic composition, and functional potential (15). These approaches have revealed that CKD is frequently associated with a disruption in the composition, diversity, and ecological balance of the intestinal microbiota (4). Although no universal definition of dysbiosis exists, recent consensus recommendations emphasize deviations from a healthy microbial ecosystem characterized by alterations in microbial diversity, community structure, and functional capacity (16). Such changes are commonly assessed using measures of α-diversity, which quantify within-sample microbial richness and evenness, and β-diversity, which evaluates differences in microbial community composition between samples (17). Taxonomic analyses spanning multiple hierarchical levels, including phylum, class, order, family, genus, and species, have demonstrated that CKD is associated with distinct microbial signatures compared with healthy individuals (18).

Given the alterations in gut microbial composition observed in CKD, increasing attention has focused on modifiable factors that may influence the intestinal microbial ecosystem, including dietary approaches. Among these, dietary fiber is of particular interest, as inadequate fiber intake is highly prevalent in individuals with CKD (6, 19). Several factors contribute to this inadequate intake, including dietary restrictions aimed at managing hyperkalemia and hyperphosphatemia, which often limit the primary sources of dietary fiber, such as fruits, vegetables, legumes, nuts, and whole grains(20). Reduced appetite, dietary monotony, and concerns regarding mineral intake may further exacerbate insufficient fiber consumption (20). Consequently, fiber intake among patients with CKD frequently falls below recommended levels, potentially contributing to alterations in gut microbial community structure and function (21). Dietary fiber has, therefore, emerged as a promising nutritional strategy for modulating the gut microbiome in CKD. However, dietary fibers are structurally diverse and differ substantially in their physicochemical properties, including fermentability, and viscosity (17). Fermentability refers to the extent to which a fiber can be utilized by gut microorganisms as a substrate for growth and metabolism, influencing microbial abundance, diversity, and community composition (22). Viscosity describes a fiber’s capacity to form a paste or gel when combined with water and increase the thickness of intestinal contents, which may alter microbial access to substrates and modify ecological conditions within the gut (23). Together, these properties influence how individual fibers interact with the gut microbiome and may contribute to the heterogeneous responses observed across fiber intervention studies. Consequently, the effects of dietary fiber on gut microbial composition are unlikely to be uniform, highlighting the importance of considering fiber-specific characteristics when evaluating microbiome-targeted interventions in CKD (24).

Despite increasing interest in dietary fiber as a strategy for modulating the gut microbiome in CKD, the current evidence remains fragmented. Clinical trials have evaluated a wide range of fiber interventions that differ substantially in fiber type, dose, duration, study population, and microbiome assessment methods, limiting direct comparisons across studies. In addition, previous reviews have primarily focused on broader clinical outcomes or have examined fiber interventions without considering the physicochemical properties of the fibers administered. Consequently, important questions remain regarding whether specific fiber characteristics influence gut microbial composition differently and the extent to which these effects are consistent across CKD populations. Given that dietary fibers vary considerably in their fermentability and viscosity, understanding how these properties shape microbial community structure is critical for interpreting the heterogeneous findings reported in the literature. To address these knowledge gaps, this systematic review synthesizes evidence from human and animal studies investigating the effects of dietary fiber interventions on gut microbiome composition in CKD. Furthermore, we evaluate whether fiber fermentability and viscosity are associated with differential changes in microbial diversity and taxonomic composition. By integrating microbiome outcomes with fiber physicochemical characteristics, this review aims to provide a framework for understanding how specific fiber types may influence the gut microbiome in CKD and to identify priorities for future research and dietary intervention studies.

## Methods

This systematic review and meta-analysis was conducted according to the methodological standards described in the Cochrane Handbook for Systematic Reviews of Interventions for human studies and SYRCLE’s guide for systematic reviews of preclinical animal intervention studies (25). The review was reported in accordance with the Preferred Reporting Items for Systematic Reviews and Meta-Analyses (PRISMA) 2020 guidelines (26). The protocol was prospectively registered in the International Prospective Register of Systematic Reviews (PROSPERO; registration number CRD42023483468). The study design was guided by the PICOS framework (Population, Intervention, Comparison, Outcomes, and Study design) (27), as outlined in Table 1.

### Eligibility criteria

Studies were eligible if they were randomized controlled trials (RCTs) in humans or controlled experimental studies in animal models that evaluated the isolated effects of dietary fiber in the context of CKD. Eligible human populations included adults (≥ 18 years) with CKD at any stage, including both pre-dialysis and dialysis populations. Eligible animal studies included established CKD models, such as Cy/+ rats, adenine-induced CKD, subtotal or 5/6 nephrectomy models, and *Col4A3* knockout models. Animal models incorporating CKD-relevant comorbidities, including streptozotocin-induced type 1 diabetes and hypertension models (e.g., Dahl salt-sensitive rats and leptin receptor–deficient models), were also considered. Studies conducted in healthy populations, pediatric populations, or models of acute kidney injury were excluded. Interventions were required to consist of a single, well-defined dietary fiber supplement to allow characterization of physicochemical properties such as fermentability and viscosity. Fiber provided as part of whole foods, mixed diets, or multi-component interventions was excluded unless the independent effect of the fiber component could be clearly isolated. No restrictions were applied based on geographic region; however, the search was limited to articles published in English.

Studies were eligible if they included a comparator arm, defined as a standard diet, control condition, or placebo; single-arm studies without a comparator group were excluded. Eligible studies were also required to report at least one sequencing-derived measure of gut microbial composition from fecal or cecal samples, including α-diversity indices, β-diversity metrics, or the relative abundance of microbial taxa at any taxonomic rank. Only studies providing sufficient quantitative or directional data to allow interpretation of changes in microbial composition were considered. *In vitro* studies, observational designs (including cohort and case-control studies), case reports, case series, and review articles were excluded. Due to substantial heterogeneity in study designs, sequencing methodologies, bioinformatics pipelines, and reporting of microbiome outcomes, quantitative synthesis was not feasible. Differences in targeted 16S rRNA regions, sequencing platforms, and taxonomic classification approaches limited comparability across studies. Therefore, a qualitative synthesis was undertakento integrate the findings from methodologically diverse studies, allowing for meaningful interpretation of overall patterns and trends in gut microbiota composition (See Table 2).

### Literature search

A systematic literature search was carried out on 15 November 2023 by a research librarian (JBR) and a PhD candidate (SNM), with an update performed on 6 February 2024 that identified one additional randomized controlled trial. Four electronic databases were searched: PubMed, Embase (via Elsevier), CINAHL (via EBSCO), and the Cochrane Library (including CENTRAL and Cochrane Reviews). In addition, the reference lists and included studies of relevant review articles identified through the search were hand-searched to identify any eligible studies not captured by the electronic database search. The search strategy combined controlled vocabulary with relevant keywords covering chronic kidney disease, dietary fiber, gut microbiota, fermentable and non-fermentable fibers, and microbial composition. To ensure comprehensive coverage, reference lists of included studies and relevant review articles were manually examined, and citation tracking was conducted to identify any additional eligible studies. The complete search strategies for all databases are provided in the **Supplementary Document**.

### Study selection and data extraction

All search results were imported into Covidence (28), for deduplication and screening. Screening was conducted in two stages: first, titles and abstracts were independently assessed by two reviewers (AB and SNM); subsequently, the full texts of potentially eligible studies were independently evaluated by the same reviewers. Any discrepancies were resolved through discussion or, when needed, consultation with a senior reviewer (BK). Data extraction was performed using a standardized Excel template and completed independently by three reviewers (SNM, CC, and HEW). The search strategy was intentionally broad to capture studies investigating both gut microbiota composition and gut-derived microbial metabolites in relation to CKD and dietary fiber interventions. Due to the scope of the retrieved studies, two separate systematic reviews were planned: one addressing microbiota composition and the other focusing on microbial metabolites. For the present review, inclusion was restricted to studies where gut microbiome composition was the primary outcome. Extracted data included study identifiers, country, design, population characteristics, sex distribution, health status, intervention duration, sample sizes, type and dose of fiber intervention, control conditions, and methods used for microbiome sequencing and taxonomic classification. For animal studies, additional details such as the animal model and CKD induction method were also recorded. When required data were missing or unclear, corresponding authors were contacted for clarification. Studies lacking essential information and without author response were excluded (See Tables 3a-b).

### Quality assessment

Due to the heterogeneity in study methodologies and the absence of raw sequencing data, a meta-analysis was not feasible. Instead, a structured qualitative assessment of microbial composition was conducted. This approach applied a system of evidence level criteria (Tables 4 and 5), based on a previously established ranking framework (29), which considers the number of studies, their methodological quality, and the consistency of findings. Snelson et al. used the Newcastle-Ottawa Scale (NOS) which is specifically designed for assessing the quality of non-randomized human studies and has been widely used to classify studies based on a numerical scoring system (with scores ≥ 7 typically considered high quality). In the present study, however, methodological quality was assessed using risk of bias (RoB) tools tailored to study design. For human studies, a 5-domain Cochrane RoB tool was applied, which provides an overall risk of bias judgment; studies were classified based on this overall assessment(30). For animal studies, the SYRCLE risk of bias tool was used, which consists of 10 domains and is specifically adapted for preclinical experimental research. As SYRCLE does not generate an overall score, given that summarizing across domains may obscure the relative importance of individual biases, study quality was not reduced to a single rating (25). Instead, the distribution of low, unclear, and high risk judgments across domains was considered. However, the visualization system required an overall score field and did not allow this field to be excluded; therefore, it was retained but left blank.

To ensure consistency with established evidence grading approaches, the classification of “high-quality” studies using RoB tools was aligned conceptually with NOS-based thresholds. For animal studies assessed using SYRCLE, studies were considered lower risk of bias when the majority of domains were rated as low risk, with minimal high-risk judgments. For human studies, the overall RoB judgment was used directly for classification. Findings were considered highly consistent and graded as ‘strong evidence’ when ≥ 75% of studies reporting a specific bacterial outcome showed agreement in the direction of effect (increase or decrease relative to controls), including at least two studies classified as low risk of bias. ‘Moderate evidence’ was defined as consistent findings in ≥ 75% of studies, including one low-risk study and at least one study with higher or unclear risk. ‘Weak evidence’ was assigned when evidence was based on a single low-risk study or multiple studies with higher or unclear risk. Findings were considered ‘inconclusive’ when results were inconsistent or when an insufficient number of studies were available (Table 5). Finally, data on the functional potential of the microbial community were qualitatively summarized.

## Results

**Figure 1:**
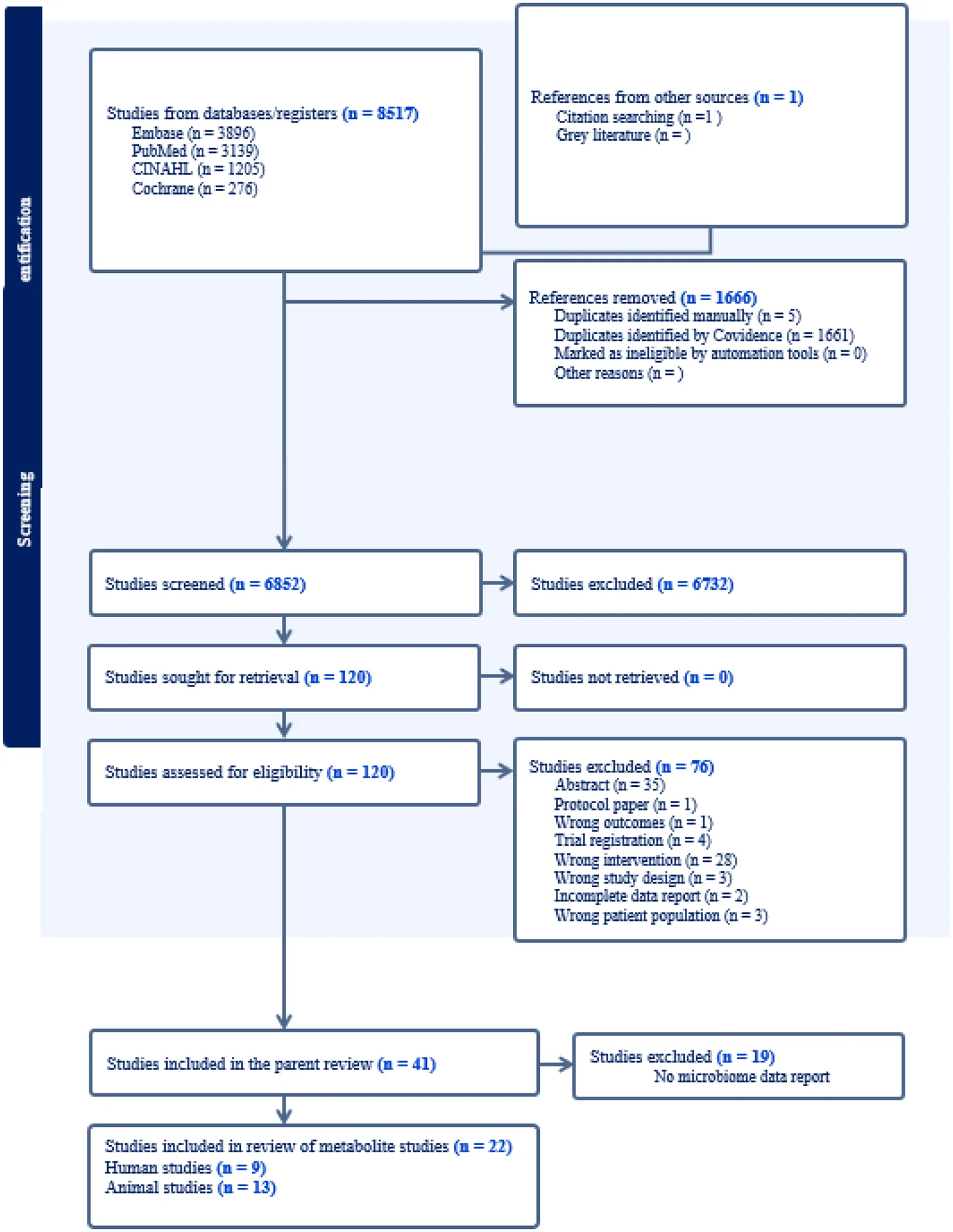
PRISMA flow chart of the study selection process for inclusion studies in the systematic review.

The study selection process identified 8,517 records through database searches, along with one additional study from citation tracking. After removing 1,666 duplicates (5 manually and 1,661 via Covidence), 6,852 records remained for screening, of which 6,732 were excluded based on title and abstract. A total of 120 articles underwent full-text assessment, resulting in the exclusion of 76 studies due to abstract-only publications (n = 35), incorrect intervention (n = 28), trial registrations (n = 4), inappropriate study design (n = 3), incorrect population (n = 3), protocol papers (n = 1), incomplete data (n = 2), and irrelevant outcomes (n = 1). Ultimately, 22 studies, 9 human (31–40) and 13 animal studies (41–53) met, the inclusion criteria and were included in the final systematic review (See Fig. 1).

### Basic characteristics of human studies

The systematic review of 9 included RCTs (31, 32, 34–40) encompassing a total of 256 participants, with 129 assigned to intervention groups and 127 to control groups; some participants contributed to both groups due to crossover study designs. Most trials were conducted in the USA (n = 3) (31, 34, 36) and China (n = 2) (35, 40) with additional studies carried out in Brazil (n = 1) (37), Iran (n = 1) (38), Italy (n = 1) (39) and South Africa (n = 1) (32). All included studies were randomized and controlled, employing either parallel or crossover designs. Study populations consisted of individuals with CKD, some undergoing dialysis (31, 33–35, 37, 38, 40). Trial durations varied from 4 to 24 weeks, with the majority lasting between 4 and 8 weeks. Mean participant ages ranged from 18 to 77 years, and all studies included both male and female participants. Interventions involved supplementation with various dietary fibers, including inulin, resistant starch type 2, and β-glucan administered at doses ranging from 3 to 33 g/day. Control interventions included maltodextrin, waxy corn starch (AMIOCA), and wheat starch combined with a low-protein diet, with doses ranging from 10 to 20 g/day. Three studies did not include a placebo control (32, 34, 39) (See Table 3a).

### Basic characteristics of animal studies

The meta-analysis included 13 animal studies, five of which contained two distinct intervention arms, yielding 20 separate comparisons (41–53). A total of 274 rodents were included, with 161 allocated to intervention and 113 to control groups. Three studies contributed two independent comparisons due to distinct intervention arms: CKD stage-specific groups in Biruete et al. (43); two fiber doses in Yang et al. (52) and two distinct fiber types in Hung and Suzuki et al. (46); and two studies contributed to three independent studies due to distinctive arms: three fiber doses in Wang et al. (51) and three distinct fiber types (42). Most trials were conducted in USA (n = 4) (43, 47, 52, 53) and Japan (n = 3) (44, 46, 50) with additional studies performed in China (n = 3) (45, 48, 51), the Oman (n = 2) (41, 48), Thailand (n = 1) (42). Study populations consisted of rat or mouse models of CKD and diabetic kidney disease (DKD). Different mouse and rat strains were used across the studies, including the Cy/+ rat model, Wistar rats, Sprague Dawley rats, mice from the Institution of Cancer Research, and BKS Cg Lepr db mice. CKD was induced using various approaches, including progressive kidney disease models, adenine-induced injury, streptozotocin-induced diabetes, 5/6x nephrectomy-induced CKD, and a homozygous mutation in the leptin receptor gene. Some CKD models were induced at the same time as the intervention period, while others were established before the start of the intervention. Sample sizes ranged from 8 to 24 animals per study, with intervention and control groups generally balanced. Study durations varied between 2 and 12 weeks. The age of animals ranged from 6 to 22 weeks at baseline, and most studies included just male animals with one study conducted exclusively in female animals (48). Interventions involved supplementation with various dietary fibers, including inulin, xylooligosaccharides (XOS), resistant starch type 2 (RS2), psyllium husk, guar gum, gum acacia, partially hydrolyzed guar gum (PHGG), chitosan oligosaccharide (COS), galacto-oligosaccharides (GOS), and oligosaccharide-enriched inulin, administered at concentrations ranging from 1.6% to 59% (w/w). Control groups received no placebo, except for one study that used cellulose (43) and two used amylopectin (47, 53) (see Table 3b).

### Risk of Bias Assessment of Human and Animal Studies

**Figure 2:**
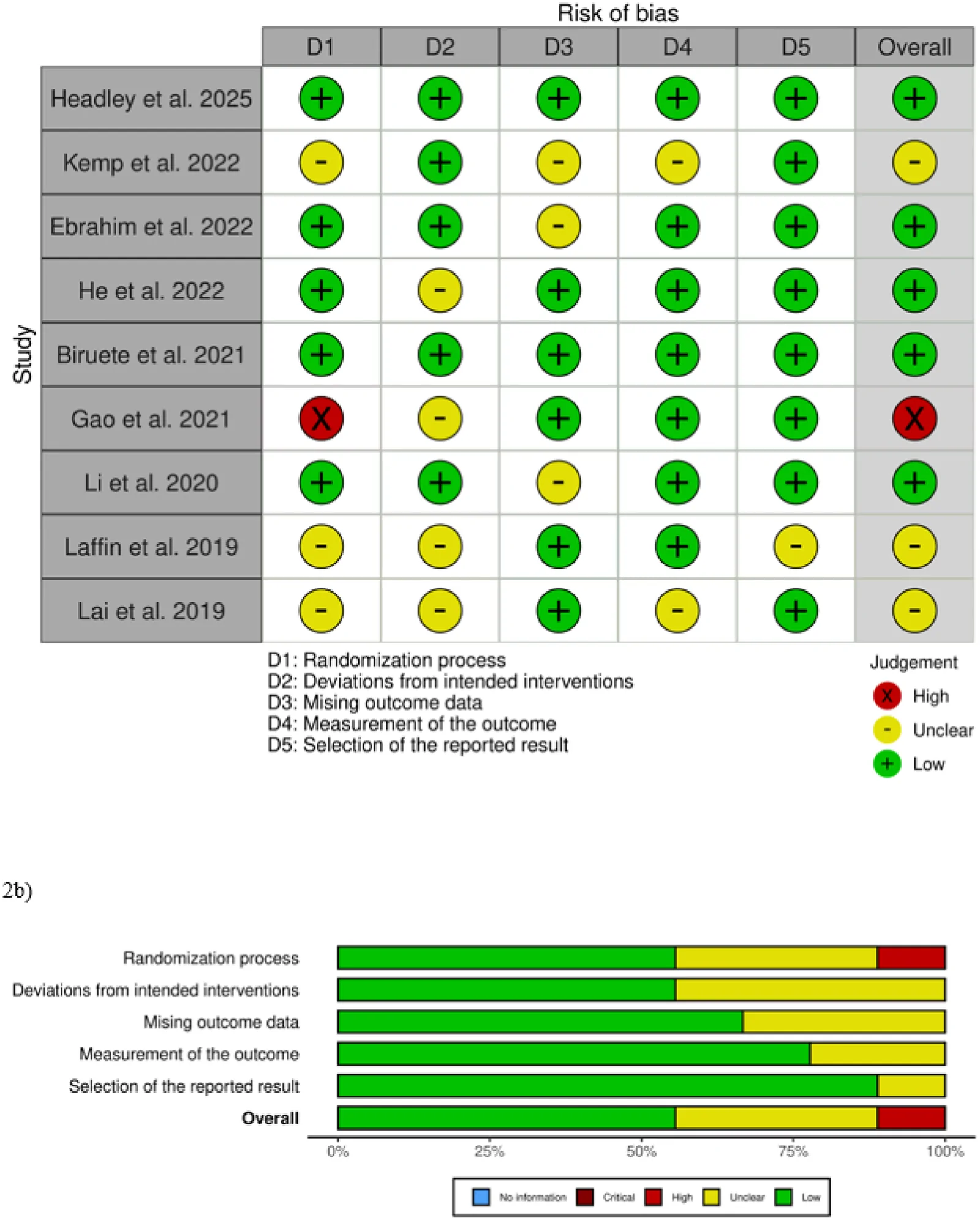
Quality assessment of human studies according to Cochrane risk-of-bias 2 (RoB-2) a) Traffic light plot, b) Summary plot

**Figure 3:**
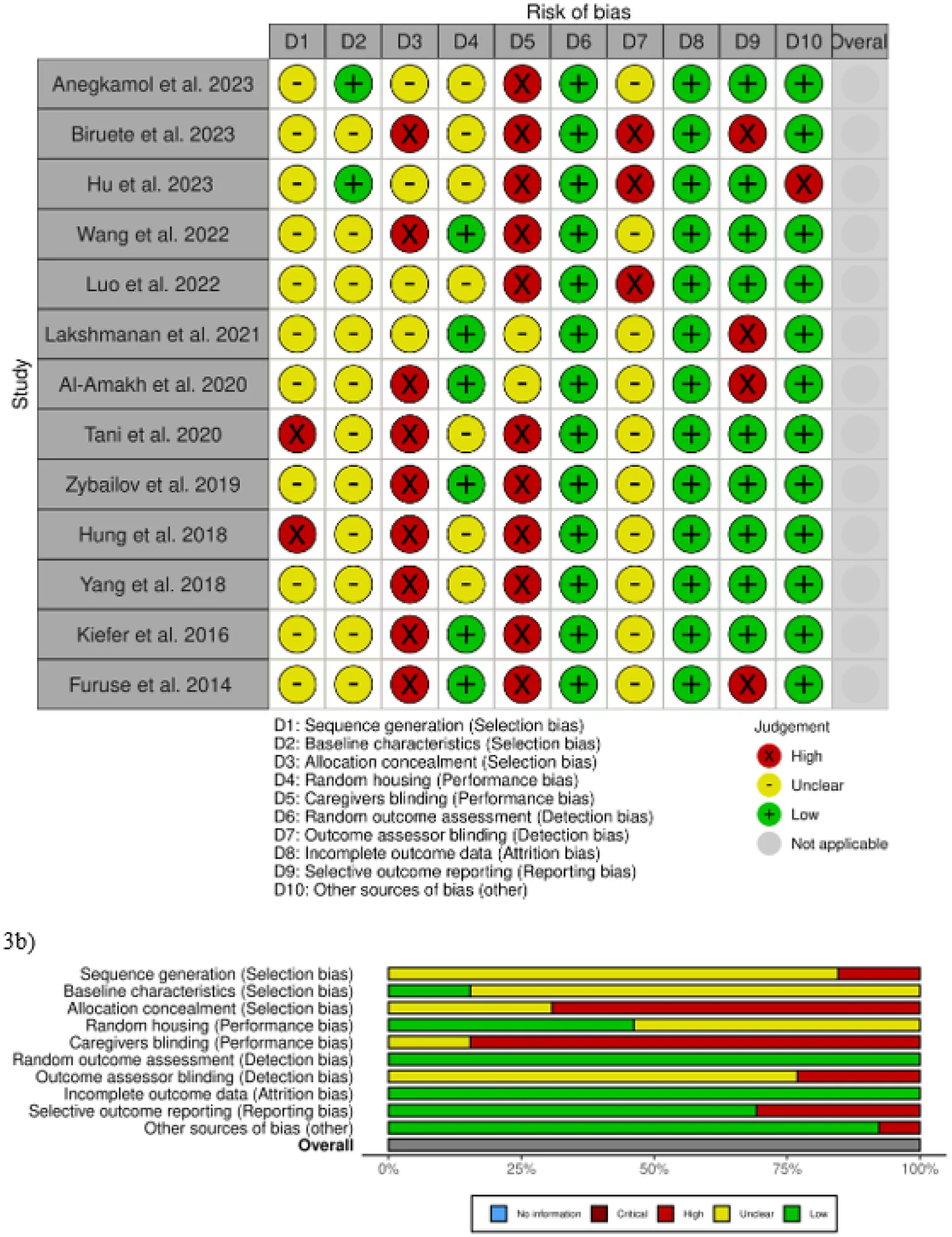
Quality assessment of animal studies according to SYRCLE’s Risk of Bias a) Traffic light plot, b) Summary plot. Because the SYRCLE risk-of-bias tool does not recommend calculating an overall score, each domain was assessed separately. However, the visualization system required an overall score field and did not allow this field to be excluded; therefore, it was retained but left blank.

Risk of bias was assessed separately for human and animal studies. Overall, the majority of human trials were judged to be at low risk of bias based on Cochrane’s risk-of-bias tool (RoB 2) for RCTs (30), with the exception of four studies (34, 37–39), which were rated as having some concerns or a high risk of bias (Figs. 2a–b). For animal studies, the risk of bias assessment using, risk of bias tool SYRCLE (25), indicated that most trials demonstrated some concerns or high risk of bias across evaluated domains, reflecting generally low methodological quality based on the assessment tool (Figs. 3a–b). Most animal studies provided limited methodological detail, resulting in unclear or high risk of bias across multiple domains. Specifically, randomization procedures and blinding of caregivers or outcome assessors were rarely described, and baseline characteristics were generally not reported or accounted for in the analyses. Allocation concealment was also not documented in nearly all studies. Overall, the reporting quality of animal studies was insufficient to fully assess potential sources of bias.

### Gut Microbiota Changes Following Dietary Fiber Supplementation

Results are organized according to changes in gut microbial composition, presented first for human studies and then for animal studies. Within each model, microbial diversity and taxonomic outcomes are reported in relation to the corresponding dietary fiber intervention and fiber classification, with comprehensive study-level details provided in Tables 8a and 8b. These tables summarize changes in α-diversity, β-diversity, and taxonomic composition at the phylum, order, family, and genus levels. Fiber interventions are presented by both fiber name (e.g., inulin) and physicochemical classification (i.e., fermentable/non-fermentable and viscous/non-viscous). Direction of change is color-coded in the tables, with increases shown in green, decreases in orange, and no changes shown in grey. For β-diversity, significant differences in microbial community composition are shown in blue. Each cell reports the number of studies corresponding to the observed result.

## HUMAN STUDIES

### Microbial diversity (α- and β-diversity) changes according to dietary fiber viscosity and fermentability

Alpha (α)-diversity, reflecting within-sample microbial diversity, was assessed using richness measures (Observed Features and Chao1), evenness (Pielou’s evenness), and composite diversity metrics (Shannon and Simpson indices). Two studies evaluated α-diversity and reported no significant effects of inulin on Observed Features and Shannon index or β-glucan on the Shannon index and observed features compared with controls (32, 34). These findings suggest that neither fermentable non-viscous fiber, such as inulin, nor fermentable viscous fiber, such as β-glucan, consistently altered within-sample microbial diversity.

Beta (β)-diversity, reflecting between-sample differences in microbial community composition, was assessed using abundance-based (Bray–Curtis), presence/absence-based (Jaccard), and phylogeny-based measures of community dissimilarity, including weighted and unweighted UniFrac distances. Three studies evaluated β-diversity, with inconsistent findings across fiber types. RS2, a fermentable non-viscous fiber, and β-glucan, a fermentable viscous fiber, showed no significant effects on Bray-Curtis or weighted UniFrac distances, respectively (32, 36). In contrast, inulin, also classified as fermentable and non-viscous, significantly altered β-diversity based on weighted UniFrac (39). Overall, fermentability and viscosity alone did not reliably predict changes in microbial diversity or community structure, suggesting that fiber source, dose, intervention duration, study design, and baseline microbiome composition may also contribute to the observed variability.

### Taxonomic changes:abundances of phylum-level taxa according to dietary fiber viscosity and fermentability

Phylum-level taxonomic outcomes were reported only for inulin interventions, as other fiber studies did not assess or report results at this taxonomic level. Across three studies, the effects of inulin supplementation were inconsistent and yielded few significant between-group differences, and the evidence-ranking system largely supported null effects at the phylum level (31, 35, 40). Among the three studies evaluating Bacillota (formerly Firmicutes), two reported no significant differences compared with control interventions (31, 40), whereas one study observed a significant increase following inulin supplementation (35). This increase was reported in a 12-week crossover trial of 16 non-diabetic adults with stage 3–4 CKD receiving continuous ambulatory peritoneal dialysis and consuming 10 g/day of inulin (35). Despite this increase, the overall evidence was ranked as strong for no change in Bacillota because two studies reported null effects. Three studies assessed Bacteroidota (formerly Bacteroidetes) and generally reported no significant between-group differences (31, 40) with the exception of the same study by He et al. (35). Accordingly, the evidence was ranked as strong for no change in Bacteroidota. Similarly, no significant effects of inulin were observed for Pseudomonadota, formerly Proteobacteria, across three studies (31, 35, 40), supporting strong evidence for no change, or for Fusobacteriota in one study (40), providing weak evidence for no change. In contrast, a study reported significant increases in Verrucomicrobiota following inulin supplementation, providing weak evidence for an increase; which was a 4-week crossover trial in 12 hemodialysis patients receiving sex-specific doses of inulin, 10 g/day for females and 15 g/day for males (31). Notably, significant phylum-level alterations were observed only in the study using shotgun metagenomic sequencing. In contrast, studies using 16S rRNA gene sequencing generally reported no significant phylum-level effects, with the exception of an increase in Verrucomicrobiota (31). Overall, these findings suggest that the phylum-level effects of inulin are variable and may be influenced by sequencing approach, CKD population, dialysis status, dose, intervention duration, and baseline microbial composition.

### Taxonomic changes:abundances of order and family-level taxa according to dietary fiber viscosity and fermentability

Order- and family-level taxonomic outcomes were reported primarily for fermentable non-viscous fibers, particularly inulin, which was evaluated in three studies (31, 39, 40). One study also reported family-level outcomes for RS2 (37), whereas other fiber interventions did not assess or report results at these taxonomic levels. Across studies, the effects of inulin were inconsistent. At the order level, inulin increased Clostridiales in one study (35), whereas no significant change was observed in another study (31). Notably, the former used shotgun metagenomic sequencing, while the latter used 16S rRNA gene sequencing. At the family-level, inulin increased Bacteroidaceae, Bifidobacteriaceae, and Rickenellaceae in one study (39), although Rickenellaceae was unchanged in another study (31), providing weak evidence for no change in these families. Findings for Enterobacteriaceae were also inconsistent, with one study reporting a significant decrease (39) and two studies reporting no significant change (31, 40). Inulin did not significantly alter Erysipelotrichaceae or Lachnospiraceae in one study (31). For RS2, no significant change in Prevotellaceae was reported at the family level (37). Overall, order- and family-level findings were limited and inconsistent, suggesting that fiber-specific effects at lower taxonomic levels may depend on sequencing approach, sample type, study design, and baseline microbial composition.

### Taxonomic changes:abundances of genus-level taxa according to dietary fiber viscosity and fermentability

Genus-level taxonomic outcomes were reported primarily for fermentable non-viscous fibers, particularly inulin, which was evaluated in five studies (31, 34, 35, 39, 40). Among these, two studies used shotgun metagenomic sequencing (34, 35) and three used 16S rRNA gene sequencing (31, 39, 40). Genus-level outcomes were also reported for RS2 in three studies (36–38) and for the fermentable viscous fiber β-glucan in one study (32).

Across studies, inulin produced heterogeneous genus-level responses, with several findings differing by sequencing approach. Shotgun metagenomic studies reported increases in *Anaerostipes* (34, 35), *Faecalibacterium* and *Romboutsia* (34), whereas no significant changes in these taxa were observed in a 16S rRNA-based study (40). Similarly, decreases in *Blautia*, *Dorea, Erysipelatoclostridium, Lachnoclostridium*, and *Ruminococcus* were reported using shotgun metagenomics (34), while no significant changes were reported for these genera in 16S rRNA studies (31, 40). In the same shotgun metagenomic study, both increases and decreases were observed for *Lactobacillus, Streptococcus, unclassified Lachnospiraceae*, and *Shigella* at the strain level (34); however, these genera were unchanged in 16S rRNA-based studies (31, 40). *Bacteroides* also showed inconsistent findings, with one shotgun metagenomic study reporting an increase (34) and another reporting a decrease (35), whereas no significant changes were observed in 16S rRNA studies (31, 40). Additional increases following inulin supplementation were reported for *Akkermansia* (31); *Actinomyces, Atopobium, Citrobacter, Lactococcus, Megasphaera, Proteus, Ralstonia, Raoultella, Rothia, Staphylococcus, unclassified Coriobacteriaceae, and Xenorhabdus* (34); and *Clostridium* (34, 35). Conversely, significant decreases were reported for *Coprobacillus, Enterobacter, Fournierella, Granulicatella, Merdibacter, Turicibacter*, and *unclassified Erysipelotrichaceae* (34) Several genera showed no significant response to inulin, including *Agathobacter, Butyricicoccus, Enterococcus, Escherichia-Shigella, Erysipelotrichaceae_UCG-003, Fusicatenibacter, Intestinibacter, Klebsiella, Pseudomonas, Ruminococcaceae_UCG-013, Ruminococcaceae_UCG-014, Subdoligranulum, Tyzzerella, unclassified Enterobacteriaceae*, and *Veillonella* (40); *Bifidobacterium, Collinsella, Eubacterium, Parabacteroides*, and *Phascolarctobacterium* (31, 40); and *Coprococcus, Oscillospira*, and *Pyramidobacter* (31). Among these null findings, only the lack of change in *Bifidobacterium, Collinsella, and Eubacterium* was supported by strong evidence. Overall, genus-level responses to inulin were highly variable and appeared to differ by sequencing platform, taxonomic resolution, and study context.

RS2 was associated with genus-level changes across three studies (36–38). RS2 increased *Bacteroides, Oscillospiraceae UCG-002*, and *Subdoligranulum* in one study (36), and *Faecalibacterium* in another study (38). In contrast, no significant changes were reported for *Bifidobacterium, Parabacteroides, or Ruminococcus* (38); *Bilophila, Desulfovibrio, Escherichia-Shigella, or Ruminococcaceae UCG-011* (37); or *Prevotella* (37, 38). Overall, RS2 produced selective genus-level shifts, with effects that varied across studies and appeared to be taxa-specific.

β-glucan, a fermentable viscous fiber, showed no significant genus-level effects on *Bacteroides, Faecalibacterium, Prevotella, Rothia, or Ruminococcaceae UCG-014* reported in a study (32).

## ANIMAL STUDIES

### Microbial diversity (α- and β- diversity) changes according to dietary fiber viscosity and fermentability

Alpha-diversity was assessed using measures of richness (Observed Features and Chao1), evenness (Pielou’s Evenness), overall diversity (Shannon and Simpson indices), and phylogenetic diversity (Faith’s Phylogenetic Diversity). Nine studies evaluated α-diversity, with heterogeneous findings across fiber interventions. No significant changes were reported following supplementation with inulin (42, 43, 49) resistant maltodextrin (42), or XOS (52), whereas reductions were observed with inulin (43), XOS (52), gum acacia (48), PHGG (50), and psyllium husk (45). Increases in α-diversity were reported in studies using β-glucan (51), psyllium husk (45) and RS2 (53) supplementation, although the latter was assessed using cecal metaproteomics rather than sequencing-based microbiome profiling. The conflicting findings for psyllium husk and inulin reflect differences in the α-diversity metrics assessed, with increases reported for Chao1 and Simpson’s indices and a decrease reported for the Shannon index (45). The effects of dietary fiber on α-diversity were inconsistent and appeared to vary by fiber type and study context.

Beta-diversity was assessed using weighted and unweighted UniFrac distance metrics, consistent with the approaches reported in human studies. Four studies evaluated β-diversity (43, 50–52). Significant shifts in microbial community composition were reported following supplementation with inulin (43), XOS (52), COS (51), or PHGG (50) whereas COS showed no significant effect in one study (51) depending on the β-diversity metric used. Collectively, β-diversity findings indicate that several fiber interventions altered overall microbial community structure, but these effects were not consistently predicted by fermentability or viscosity alone.

### Taxonomic changes:abundances of phylum-level taxa according to dietary fiber viscosity and fermentability

Among fermentable non-viscous fibers, phylum-level taxonomic changes were reported for inulin (42, 43, 49), RS2 (47), resistant maltodextrin (42), XOS, and COS (42). Inulin was evaluated in three studies and showed no significant effects on phylum-level composition in most comparisons (42, 49), except for a significant increase in Bacteroidota, formerly Bacteroidetes, in the cecal microbiota of CKD rats reported by Biruete et al. (43); the remaining studies assessed fecal samples. RS2 supplementation altered several phyla, including significant increases in Actinomycetota, formerly Actinobacteria, and Pseudomonadota, formerly Proteobacteria, and a significant decrease in Bacillota, formerly Firmicutes, while no significant changes were observed in Bacteroidota, Tenericutes, or Verrucomicrobia (47). Resistant maltodextrin had minimal effects at the phylum level, with no significant changes except for a decrease in Verrucomicrobia (42). Similarly, XOS supplementation did not significantly alter any assessed phyla (52). COS also showed limited phylum-level effects, with no significant changes except for an increase in Patescibacteria (42). Overall, fermentable non-viscous fibers produced heterogeneous phylum-level responses, suggesting that fiber source and experimental context, including sample type, may influence observed changes in gut microbial composition.

Among fermentable viscous fibers, phylum-level taxonomic changes were reported for gum acacia (41, 48), guar gum, and PHGG (46). Gum acacia was evaluated in two studies and showed inconsistent effects on Bacillota (formerly Firmicutes) with one study reporting a significant increase (41) and another reporting a significant decrease (48). In the latter study, gum acacia also increased Bacteroidota (formerly Bacteroidetes) and Pseudomonadota (formerly Proteobacteria) while decreasing Desulfobacterota and Verrucomicrobiota (48). Guar gum and PHGG increased Bacillota in one study (46). Overall, fermentable viscous fibers produced variable phylum-level responses, suggesting that fiber source, study design, and experimental context may influence the direction of taxonomic changes.

Among viscous, poorly fermentable fibers, psyllium husk was the only fiber evaluated at the phylum level (45). In one study, psyllium husk supplementation significantly increased Actinomycetota (formerly Actinobacteria) and significantly decreased Bacillota (formerly Firmicutes) (45).

### Taxonomic changes:abundances of order and family-level taxa according to dietary fiber viscosity and fermentability

Among fermentable non-viscous fibers, order- and family-level taxonomic changes were reported for galactooligosaccharide (GOS) (44), inulin (43), and RS2 (47). GOS showed divergent effects within the order Clostridiales, with a significant increase in Clostridiales; Incertae Sedis XIV and a significant decrease in Clostridiales; Incertae Sedis XIII (44). The same study also reported increases in Bifidobacteriaceae and Porphyromonadaceae, alongside decreases in Clostridiaceae, Peptostreptococcaceae, Ruminococcaceae, Streptococcaceae, and Veillonellaceae (44). Inulin was evaluated in three studies and was associated with an increase in Barnesiellaceae and decreases in Peptostreptococcaceae and Rickenellaceae (43). RS2 decreased the orders Bacteroidales and Clostridiales and reduced several families, including Clostridiaceae, Desulfovibrionaceae, Lachnospiraceae, Rickenellaceae, and Ruminococcaceae (47). In contrast, RS2 increased Barnesiellaceae, Coriobacteriaceae, Enterobacteriaceae, and Erysipelotrichaceae (47). No studies reported order- or family-level taxonomic outcomes for fermentable viscous fibers or viscous non-fermentable fibers.

### Taxonomic changes:abundances of genus-level taxa according to dietary fiber viscosity and fermentability

Among fermentable non-viscous fibers, genus-level taxonomic changes were reported for inulin (43, 49), RS2 (47, 53), XOS (52). Inulin supplementation increased *Akkermansia, Candidatus Saccharimonas, Mucispirillum*, and *Prevotella* (49), as well as *Allobaculum, Bacteroides, Bifidobacterium, Sutterella*, and *unclassified Lachnospiraceae* (43). Conversely, inulin decreased *Adlercreutzia, Dorea, Lactobacillus, Oscillospira, unclassified Clostridiaceae*, and *unclassified Ruminococcacea*e (43), as well as *Turicibacter* (49). RS2 increased *Allobaculum, Bifidobacterium, Catenibacterium, Eubacterium, Faecalibacterium, Parabacteroides, Paraprevotella*, and *Sutterella* (47), while decreasing *Bilophila* (53) and *Blautia, Coprococcus, Lactococcus, Lachnospira, Odoribacter, Prevotella*, and *Turicibacter* (53). *Ruminococcus* showed inconsistent responses to RS2, with both increases (47, 53) and decreases (53) reported, likely reflecting species-level differences. XOS supplementation decreased *Allobaculum* and *Dorea*, while no significant changes were observed in *Blautia, Coprobacillus*, or *Ruminococcus* (52). In contrast, responses for *Clostridium, Lactobacillus, Staphylococcus*, and *unclassified Erysipelotrichaceae* were inconsistent and appeared to vary according to intervention duration (52).

Among fermentable viscous fibers, genus-level taxonomic changes were reported for β-glucan (51), guar gum (46), and PHGG (46, 50). β-glucan showed dose-dependent effects, with higher doses increasing *Akkermansia* and *Butyricicoccus*, whereas lower doses produced no significant changes in these genera (51). In the same study, *Prevotella* decreased at higher doses but remained unchanged at lower doses (51). Guar gum increased *Lactobacillus* and *Bifidobacterium* (46). PHGG increased *Lactobacillus* (50), while decreasing *Atopobium, Bacteroides*, and *Desulfovibrio* (50), as well as *Bifidobacterium* (46).

Among viscous, poorly fermentable fibers, psyllium husk was the only fiber evaluated at the genus level (45). Psyllium husk increased *Acetatifactor, Oscillibacter, Ruminococcus, Saccharibacter*, and *Turicibacter*, while decreasing *Bacteroides, Blautia, Dorea, Methanobrevibacter*, and *Romboutsia* (45).

## HUMAN STUDIES vs. ANIMAL STUDIES

Although findings from human and animal studies may differ due to inherent differences in study design, host physiology, CKD model, fiber dose, intervention duration, and microbiome assessment methods, comparing the direction and consistency of responses across models may provide additional insight into the reproducibility of fiber-related effects on gut microbial composition. This cross-model comparison is summarized in Tables 9a-d.

### Microbial diversity (α- and β- diversity) changes according to dietary fiber viscosity and fermentability

Overall, human and animal studies showed limited consistency in α-diversity responses to dietary fiber interventions. Human studies reported no significant changes in α-diversity, whereas animal studies showed mixed findings, including increases, decreases, and null effects depending on fiber type and study context. For β-diversity, inulin was the only fiber showing concordant evidence of altered microbial community structure across both human and animal studies. However, other fibers produced divergent findings between models. Collectively, these results suggest that dietary fiber effects on microbial diversity are not consistently predicted by fermentability or viscosity alone and may depend on fiber-specific characteristics, intervention design, host physiology, and baseline gut microbial composition (**see** Table 9a).

### Taxonomic changes:abundances of phylum-level taxa according to dietary fiber viscosity and fermentability

Overall, concordance between models was limited. In human studies, phylum-level outcomes were reported only for inulin, a fermentable non-viscous fiber, and largely supported no significant changes in Actinomycetota, Bacillota, Bacteroidota, and Pseudomonadota, with weak evidence for increased Verrucomicrobiota. In contrast, animal studies included fermentable non-viscous fibers, such as inulin, RS2, resistant maltodextrin, XOS, and COS; fermentable viscous fibers, such as gum acacia, and guar gum, fermentable poorly viscous fiber such as PHGG; and a viscous poorly fermentable fiber, psyllium husk.

For Actinomycetota, human studies showed no change following inulin, whereas animal studies reported increases with RS2, a fermentable non-viscous fiber, and psyllium husk, a viscous poorly fermentable fiber. Bacillota was largely unchanged in human studies of inulin despite one reported increase, but animal studies showed divergent responses across fiber classes, including decreases with RS2 and psyllium husk and increases with fermentable viscous fibers such as guar gum/PHGG. Bacteroidota also differed across models; human studies generally supported no change with inulin, whereas animal studies reported increases with inulin in cecal samples and with gum acacia, a fermentable viscous fiber. Pseudomonadota was unchanged in human studies following inulin but increased in animal studies following RS2 and gum acacia. Verrucomicrobiota showed opposite directional patterns, increasing in one human study on inulin but decreasing in animal studies following resistant maltodextrin, a fermentable non-viscous fiber, and gum acacia. Other phyla were sparsely reported and could not be directly compared, including Fusobacteriota in human studies and Tenericutes, Desulfobacterota, and Patescibacteria in animal studies. Together, these findings suggest that phylum-level effects were more limited in human studies and more heterogeneous in animal models, with no consistent pattern attributable to fermentability or viscosity alone (**see** Table 9b).

### Taxonomic changes:abundances of order and family-level taxa according to dietary fiber viscosity and fermentability

At the order level, Clostridiales was the only taxon reported in both models, but responses differed by fiber type and taxonomic subgroup. In human studies, inulin either increased or did not alter Clostridiales, whereas animal studies showed both increases and decreases with GOS and decreases with RS2. Bacteroidales was reported only in animal studies and decreased following RS2.

At the family level, Bifidobacteriaceae was the only taxon showing a consistent direction of change across models, increasing in both human and animal studies, although with different fermentable non-viscous fibers. In contrast, Rickenellaceae, Enterobacteriaceae, Erysipelotrichaceae, and Lachnospiraceae showed divergent responses between models. Several families, including Clostridiaceae, Peptostreptococcaceae, Ruminococcaceae, Streptococcaceae, Veillonellaceae, Desulfovibrionaceae, and Coriobacteriaceae, were reported only in animal studies and therefore could not be directly compared. Overall, animal studies showed broader order- and family-level shifts, whereas human findings were more limited and inconsistent **(see** Table 9c).

### Taxonomic changes:abundances of genus-level taxa according to dietary fiber viscosity and fermentability

Overall, concordance across models was limited. *Akkermansia* showed the clearest similarity, increasing with inulin, a fermentable non-viscous fiber, in both human and animal studies, and also increasing with high-dose β-glucan, a fermentable viscous fiber, in animals. *Faecalibacterium* showed partial agreement, increasing with RS2 in both models, whereas β-glucan showed no effect in humans. *Dorea* and *Turicibacter* also showed some consistency, with decreases following inulin in both models. In contrast, *Bacteroides*, *Bifidobacterium*, *Ruminococcus*, *Lactobacillus*, *Prevotella*, *Parabacteroides*, and *Bilophila* showed divergent or model-specific responses. For example, *Bifidobacterium* was unchanged with inulin and RS2 in humans but increased with inulin, RS2, and guar gum in animals, while PHGG decreased it. Similarly, *Prevotella* was unchanged with RS2 and β-glucan in humans but increased with inulin and decreased with RS2 or high-dose β-glucan in animals. These findings indicate that fermentability and viscosity may contribute to genus-level responses, but they do not explain the direction of change consistently; sequencing method, dose, intervention duration, host model, and baseline microbiome composition likely contributed to the observed heterogeneity **(see** Table 9d).

## Discussion

### Aim and Main Findings

This systematic review evaluated the effects of isolated dietary fiber interventions, classified by fermentability and viscosity, on gut microbiota composition in human and animal models of CKD. Across 9 RCTs and 13 animal studies, fiber fermentability and viscosity did not consistently report changes in microbial diversity or taxonomic composition. Human studies provided limited phylum-level evidence, primarily for inulin, and largely supported no major changes, whereas animal studies reported broader and more variable phylum-level shifts across fiber types. At the order and family levels, concordance between human and animal studies was also limited, with increased Bifidobacteriaceae representing the most consistent finding across models. Most other taxa showed divergent or model-specific responses. Similarly, genus-level findings demonstrated limited agreement between human and animal studies, with only selected taxa, including *Akkermansia*, *Faecalibacterium*, *Dorea*, and *Turicibacter*, showing partial consistency across models. Overall, these findings suggest that the effects of dietary fiber on gut microbiota composition in CKD are not determined by fermentability and viscosity alone, but are likely influenced by fiber source, dose, intervention duration, sequencing method, host model, baseline microbiome composition, and study quality. The evidence-ranking system further indicated that strong evidence was limited, while most reported increases or decreases were supported by weak evidence due to limited replication and methodological heterogeneity across studies.

Interpretation of taxonomic changes in CKD requires caution, as bacterial taxa cannot be universally classified as beneficial or detrimental. The health relevance of a given taxon is highly context dependent and may vary according to host factors, CKD stage, dialysis status, diet, medication use, intestinal transit time, and the functional capacity of the microbial community (54). This is consistent with emerging evidence suggesting that host-related factors may shape gut microbiome alterations in CKD more strongly than kidney function alone (55). Moreover, microbial composition does not necessarily reflect microbial function, because functionally redundant taxa may perform overlapping ecological or metabolic roles. Therefore, changes in relative abundance should be interpreted alongside the broader host and microbial context rather than as inherently favorable or unfavorable (56).

In healthy adults, the gut microbiota is typically dominated by five major phyla, with Bacillota, formerly Firmicutes, including genera such as *Lactobacillus*, *Ruminococcus*, *Faecalibacterium*, and *Roseburia*, and Bacteroidota (formerly Bacteroidetes), including *Bacteroides* and *Prevotella*, collectively comprising the majority of the intestinal microbial community (57). These are followed by Actinomycetota (formerly Actinobacteria), which includes *Bifidobacterium*; Verrucomicrobiota, which includes *Akkermansia muciniphila*; and Pseudomonadota (formerly Proteobacteria), which includes genera such as *Escherichia* and *Salmonella* (58). However, the relative abundance and biological relevance of these taxa are influenced by multiple factors, including diet, disease state, gut transit time, microbial interactions, and strain-level functional differences. For example, comparisons between individuals with CKD and healthy controls have reported lower abundance of *Faecalibacterium* in CKD (58), which the present systematic review found that fermentable non-viscous fibers, including inulin and RS2, could increase *Faecalibacterium* in some studies. These findings suggest that selected CKD-associated microbial alterations may be modifiable through dietary fiber interventions. Nevertheless, some compositional changes observed in animal studies appeared to move taxa in directions that could theoretically counter CKD-associated perturbation, but these findings were not elaborated further because of important differences between animal models and human CKD, including host physiology, CKD induction method, sample type, fiber dose, and microbiome assessment approach.

Prebiotic fibers such as FOS, GOS, and inulin-derived oligosaccharides are commonly proposed to modulate the gut microbiome by selectively promoting saccharolytic taxa, particularly *Bifidobacterium* and *Lactobacillus* (59). Similarly, the findings of a recent study further support this concept, showing that dietary fiber increased SCFA-associated taxa, including *Faecalibacterium*, *Lactobacillus*, and *Bifidobacterium* (60). Findings from the present systematic review only partially support this expected pattern in CKD. In animal studies, fermentable non-viscous fibers, including inulin and RS2, were associated with increases in *Bifidobacterium*, consistent with the established bifidogenic effects of these substrates. However, this response was not observed consistently in human studies, where inulin and RS2 showed no significant effect on *Bifidobacterium*. In contrast, fermentable viscous fibers showed more variable effects; guar gum increased *Bifidobacterium*, whereas fermentable poorly viscous fibers such as PHGG decreased this genus in animal models. Findings for *Lactobacillus* were similarly inconsistent. Human studies reported mixed or null effects following inulin supplementation, while animal studies showed decreases with fermentable non-viscous fibers such as inulin and XOS. Conversely, fermentable viscous fibers, including guar gum and PHGG, were associated with increases in *Lactobacillus* in animal studies.

### Possible Underlying Mechanisms

Although taxonomic composition provides important insight into CKD-associated gut perturbation, the functional capacity of the gut microbiome may be more biologically relevant than microbial abundance alone. Distinct microbial communities can perform overlapping metabolic functions, a concept known as functional redundancy; therefore, compositional shifts do not necessarily translate directly into differences in microbial activity or metabolite production (61). In CKD, this distinction is particularly important because impaired kidney function leads to the accumulation of gut-derived uremic solutes, including indoxyl sulfate (IS), p-cresyl sulfate (pCS), and trimethylamine N-oxide (TMAO), which have been associated with cardiovascular complications, inflammation, and further loss of kidney function (62). This creates a self-reinforcing cycle in which declining renal clearance increases circulating uremic toxin concentrations, while toxin accumulation may further contribute to cardiovascular injury, kidney fibrosis, and CKD progression (63).

The gut microbiome is directly involved in uremic toxin accumulation. As kidney function declines, uremia induces adaptive changes in the colon, partially shifting excretory burden from the kidney to the intestinal epithelium. Urea, uric acid, and oxalate may be secreted into the intestinal lumen, where urea can be hydrolyzed by bacterial urease into ammonia and subsequently converted to ammonium hydroxide (64). This process increases luminal pH and may favor the expansion of urease-positive and proteolytic microorganisms while reducing saccharolytic bacteria involved in carbohydrate fermentation and SCFA-associated microbial profiles (65). In this altered intestinal environment, prolonged transit time, reduced fiber availability, increased luminal urea, and changes in pH act together to reshape microbial composition and function (66, 67). Thus, the relative balance between saccharolytic and proteolytic microbial activity is unlikely to be driven by a single factor; rather, it reflects the combined influence of diet, kidney function, intestinal transit, microbial substrate availability, and the uremic milieu (58).

Uremia may also compromise intestinal barrier integrity, allowing translocation of bacteria, bacterial products, and microbial metabolites into the systemic circulation (68). This can activate innate and adaptive immune responses, increase pro-inflammatory cytokine production, and contribute to adverse outcomes, including kidney fibrosis and cardiovascular complications (69). Experimental evidence further supports a causal role for specific microbial taxa in CKD progression. For example, *Eggerthella lenta* and *Fusobacterium nucleatum* have been shown to increase uremic toxin production and promote kidney disease progression in a CKD rat model, whereas supplementation with *Bifidobacterium animalis* reduced the abundance of these taxa, lowered toxin concentrations, and attenuated disease severity (70). They are also consistent with our findings that fermentable non-viscous fibers increased *Bifidobacterium* in animal studies, supporting the potential of dietary fiber supplementation to promote microbial profiles associated with more favorable gut-kidney axis outcomes.

Taken together, CKD-associated gut perturbation should be interpreted as a multifactorial process rather than a simple shift in “beneficial” or “detrimental” taxa. Cardiovascular disease risk, presence and/or severity of diabetes mellitus, constipation, delayed intestinal transit, gut permeability, systemic immune activation, dietary restrictions, reduced fiber intake, polypharmacy, dialysis status, and disease severity may all influence the gut microbiome while simultaneously being affected by microbial activity (54). Therefore, dietary fiber interventions may modify gut microbial composition and function by increasing substrate availability for saccharolytic bacteria, but their effects are likely shaped by the broader CKD environment. This complexity may partly explain why changes in microbial composition were inconsistent across studies in this review and why fermentability and viscosity alone did not fully predict microbiome responses.

### Implications for Clinical Practice

The present findings support the concept that dietary fiber may serve as a microbiome-directed nutritional tool in CKD, although the current evidence does not yet identify a specific fiber profile that reliably reshapes microbial communities. In CKD care, nutrition recommendations often focus on limiting nutrients such as potassium, phosphorus, sodium, and protein. While clinically necessary in selected patients, this approach can reduce consumption of plant foods that provide fermentable substrates for intestinal microbes. This is particularly relevant because many foods commonly restricted in CKD, including legumes, whole grains, fruits, vegetables, and nuts, are also important contributors to dietary fiber intake.

Rather than viewing dietary fiber as a single exposure, the findings of this review indicate that microbial responses should be interpreted in relation to fiber source, structure, and host context. Fibers classified as fermentable or viscous did not produce uniform effects on α-diversity, β-diversity, or taxonomic abundance across phylum, family, and genus levels. Similar fiber classes produced different outcomes across studies, and the same taxa often responded differently depending on the model, sequencing platform, sample site, dose, and intervention length. One possible explanation is that differences in baseline gut microbial community structure create distinct competitive environments for fiber utilization across individuals. This may be especially important for relatively nonspecific fermentable fibers, such as inulin, which can be metabolized by multiple microbial groups and therefore promote different taxa depending on the starting community. Overall, these inconsistencies suggest that the intestinal microbiome in CKD is shaped by a complex interaction among substrate availability, kidney disease severity, dialysis status, gut transit, medication exposure, background diet, and baseline microbial community structure.

These observations have important implications for research and clinical translation. Current CKD dietary guidance provides limited practical direction on how to incorporate fiber-rich foods or isolated fiber supplements in a way that targets microbial composition. Future trials should therefore move beyond reporting the supplement dose alone and provide detailed information on habitual fiber intake, achieved total fiber intake, fiber source, fermentability, viscosity, and food matrix. Studies should also apply standardized sequencing and bioinformatic pipelines, report taxonomic outcomes consistently, and consider CKD stage and dialysis status when interpreting microbial responses. Such work is needed to determine whether specific dietary fiber strategies can reproducibly modify gut microbial composition and whether those compositional changes are relevant to clinical outcomes in CKD.

### Study strengths and Limitations

This systematic review has several notable strengths. To our knowledge, this is the first study to synthesize both human and animal evidence on the effects of isolated dietary fiber interventions on gut microbiota composition in CKD based on fermentability and viscosity. The inclusion of both human and animal studies allowed comparison of microbial responses across models and provided insight into the translational consistency of fiber-related effects. Although human and animal studies differed in study design, host physiology, CKD model, sample type, and intervention conditions, comparing findings across models helped identify taxa with potentially consistent responses as well as taxa showing model-specific effects. Importantly, this review focused specifically on isolated dietary fibers, reducing confounding from whole-diet interventions and allowing a more targeted evaluation of fiber-specific effects. Another strength was the classification of fibers according to fermentability and viscosity, which enabled assessment of whether these physicochemical properties were associated with changes in microbial diversity and taxonomic composition at multiple levels, including phylum, order, family, and genus. This approach highlighted that fermentability and viscosity alone did not consistently explain microbial responses, emphasizing the importance of fiber source, dose, intervention duration, and study context.

Several limitations should also be acknowledged. First, there is no single gold-standard method for assessing gut microbiota composition across studies, and the included studies used different sequencing and analytical approaches, including 16S rRNA gene sequencing and shotgun metagenomic sequencing in both human and animal studies. These methods differ in taxonomic resolution and detection capacity, which may have contributed to inconsistent findings across studies. Second, sample type varied, particularly in animal studies, where both fecal and cecal samples were analyzed. Because microbial communities differ by intestinal location, combining or comparing fecal and cecal findings may limit direct interpretation. Third, reporting was incomplete and inconsistent across studies; some studies reported only selected taxa rather than comprehensive taxonomic profiles, and several taxa were evaluated in only one or a small number of studies. This limited replication made it difficult to draw firm conclusions for many fiber-taxon relationships. Fourth, human studies included heterogeneous CKD populations, including patients receiving dialysis and those not receiving dialysis, which may influence gut microbial composition because dialysis status, disease severity, diet, medication use, and uremic burden can all shape the intestinal microbiome. Finally, many animal studies had low evidence-ranking quality, and differences in CKD model, intervention dose, duration, sample collection site, and analytical pipeline further limited comparability with human studies.

Overall, while this review provides a structured synthesis of dietary fiber effects on gut microbiome composition in CKD, the evidence remains limited and heterogeneous. Future studies should use standardized microbiome methods, report full taxonomic results, distinguish fecal from cecal samples, clearly characterize fiber fermentability and viscosity, and stratify findings by CKD stage and dialysis status to clarify whether specific fiber properties consistently influence gut microbial composition.

## Conclusion

In summary, this systematic review demonstrates that isolated dietary fiber interventions can alter gut microbiota composition in human and animal models of CKD, but the direction and consistency of these changes vary substantially across studies. Fiber fermentability and viscosity did not consistently predict microbial diversity or taxonomic responses, and findings differed across taxonomic levels, host models, sequencing platforms, sample types, intervention doses, and study durations. Although selected taxa showed partial concordance across human and animal studies, most microbial responses were inconsistent or study-specific, highlighting the absence of a clear consensus in the current literature. This heterogeneity may reflect differences in study design, CKD stage, dialysis status, baseline microbiome composition, fiber source, analytical methods, and taxonomic resolution. Importantly, variation in microbial composition does not necessarily imply equivalent variation in microbial function, as functional redundancy within the gut microbiome may allow distinct microbial communities to perform overlapping metabolic roles. Therefore, even when taxonomic composition differs across studies or interventions, downstream microbial functions or metabolite profiles may not change to the same extent. Taken together, these findings suggest that dietary fiber may influence gut microbial ecology in CKD, but current evidence is insufficient to identify specific fiber characteristics that consistently predict compositional shifts. Future studies should integrate standardized microbiome sequencing, harmonized taxonomic reporting, functional profiling, and careful characterization of fiber physicochemical properties to determine whether changes in microbial composition translate into meaningful functional and clinical outcomes.

## Supplementary Material

The following supporting information can be downloaded at (). Search strategy in all databases.

Tables 3 to 9 are available in the Supplementary Files section.

Supplementary Files

This is a list of supplementary files associated with this preprint. Click to download.

- SupplementaryDocumentSystematcReviewMicrobiomePaperDisserationChapter1.docx
- Tables.docx

## Figures and Tables

**Table 1:** PICOS framework used to define eligibility criteria for the systematic review and meta-analysis. PICOS refers to Population, Intervention, Comparison, Outcomes, and Study design. Chronic kidney disease was defined according to study-specific criteria. Dietary fiber interventions were categorized based on fermentability and viscosity. Gut microbial composition

Parameter	Description
**Population (P)**	Adults with chronic kidney disease and experimental animal models of chronic kidney disease
**Intervention (I)**	Dietary fiber supplementation, classified according to fermentability and viscosity
**Comparison (C)**	No dietary fiber intervention or standard/control diet without added fiber
**Outcomes (O)**	Changes in gut microbial composition, alpha and beta diversity
**Study Design (S)**	Randomized controlled trials and experimental animal studies

**Table 2:** Eligibility criteria were defined a priori using the PICOS framework (Population, Intervention, Comparator, Outcomes, and Study design).

Domain	Inclusion Criteria	Exclusion Criteria
Population (P)	Adults (≥ 18 years) with chronic kidney disease (CKD) at any stage, including pre-dialysis or dialysis populations; experimental animal models of CKD, including Cy/+ rats, adenine-induced CKD models, subtotal/partial/5/6 nephrectomy models, and *Col4A3* knockout models; CKD-relevant comorbid animal models including type 1 diabetes (streptozotocin-induced) and hypertension models such as the Dahl salt-sensitive rat	Healthy humans or animals; children or adolescents; models of acute kidney injury
Intervention (I)	Dietary fiber provided as a single, clearly defined supplementation allowing characterization of fermentability and/or viscosity; purified fiber sources (e.g., wheat bran); prebiotic or synbiotic formulations; minimum intervention duration of ≥ 1 week	Fiber provided primarily through whole foods, fruits, vegetables, cereals, or mixed dietary patterns; multi-component interventions in which the independent effect of dietary fiber could not be discerned
Comparator (C)	Standard diet, control diet, or placebo comparator	Studies without a comparator group
Outcomes (O)	Gut microbiota composition	Studies reporting only clinical outcomes without gut microbial composition data
Study Design (S)	Original randomized controlled trials and experimental animal studies with sufficient quantitative or qualitative data (e.g., means, measures of variability, diversity indices and relative abundance)	In vitro studies; observational studies; case–control studies; case reports or case series; review articles

## Data Availability

All data used in this systematic review were extracted from published studies. The datasets supporting the findings of this study are available from the original sources cited in the manuscript. Additional information can be provided by the corresponding author upon reasonable request.

## Abbreviations

CKD: chronic kidney disease
CKD-MBD: chronic kidney disease-mineral and bone disorder
COS: Chitosan-oligosaccharide
DKD: Diabetic kidney disease
FOS: fructo-oligosaccharide
GFR: glomerular filtration rate
GOS: galactooligosaccharide
IS: indoxyl sulfate
pCS: *p*-cresyl sulfate
PHGG: partially hydrolyzed guar gum
RCT: randomized clinical trial
RS2: resistant starch type 2
SCFAs: short-chain fatty acids
SYRCLE: systematic review center for laboratory animal experimentation
TMA: trimethylamine
TMAO: trimethylamine *N*-oxide
XOS: xylooligosaccharide
